# Treatment Protocol for COVID-19 Based on T2R Phenotype

**DOI:** 10.3390/v13030503

**Published:** 2021-03-18

**Authors:** Mohamed A. Taha, Christian A. Hall, Colin J. Shortess, Richard F. Rathbone, Henry P. Barham

**Affiliations:** 1 Rhinology and Skull Base Research Group, Baton Rouge General Medical Center, 8585 Picardy Ave., Suite 210, Baton Rouge, LA 70809, USA; chall@sinusandnasalspecialists.com (C.A.H.); colin.shortess@yahoo.com (C.J.S.); richard.rathbone@gmail.com (R.F.R.); hbarham@sinusandnasalspecialists.com (H.P.B.); 2 Department of Otorhinolaryngology, Cairo University, Cairo 11451, Egypt; 3 Sinus and Nasal Specialists of Louisiana, Baton Rouge, LA 70809, USA

**Keywords:** COVID-19, bitter taste receptors, T2R38, solitary chemosensory cells

## Abstract

COVID-19 has become a global pandemic of the highest priority. Multiple treatment protocols have been proposed worldwide with no definitive answer for acure. A prior retrospective study showed association between bitter taste receptor 38 (T2R38) phenotypes and the severity of COVID-19. Based on this, we proposed assessing the different T2R38 phenotypes response towards a targeted treatment protocol. Starting July 2020 till December 2020, we tested subjects for T2R38 phenotypic expression (supertasters, tasters, and nontasters). Subjects who were subsequently infected with severe acute respiratory syndrome coronavirus 2 (SARS-CoV-2) (diagnosed via PCR) were included. Based on their taster status, supertasters were given dexamethasone for 4 days; tasters were given azithromycin and dexamethasone +/− hydroxychloroquine for 7 days; and nontasters were given azithromycin and dexamethasone for 12 days. Subjects were followed prospectively and their outcomes were documented. Seven hundred forty-seven COVID-19 patients were included, with 184 (24.7%) supertasters, 371 (49.6%) tasters, and192 (25.7%) nontasters. The average duration of symptoms with the treatment protocol was 5 days for supertasters, 8.1 days for tasters, and 16.2 days for nontasters. Only three subjects (0.4%) required hospitalization (3/3 nontasters). Targeted treatment protocol showed significant correlation (*p* < 0.05) based on patients’ T2R38 phenotypic expression. Assessing treatment protocols for COVID-19 patients according to their T2R38 phenotype could provide insight into the inconsistent results obtained from the different studies worldwide. Further study is warranted on the categorization of patients based on their T2R38 phenotype.

## 1. Introduction

A cluster of viral pneumonia cases associated with a novel Coronavirus (2019-nCoV) was first identified in Wuhan, Hubei Province, China, in December 2019 and has rapidly spread around the world, causing a global health crisis. The disease was subsequently named Coronavirus Disease-2019 (COVID-19) by the World Health Organization and has been designated Severe Acute Respiratory Syndrome-Coronavirus 2 (SARS-CoV-2). Significant concern has arisen within the global community regarding the potential risks of infectious transmission of SARS-CoV-2 [1].

The outbreak is still progressing and the pandemic is still uncontrolled, and the morbidity, mortality, and transmissibility of this novel coronavirus remain unresolved. Without the availability of effective antiviral therapies, compulsory measures have to be taken to prevent person-to-person transmission. However, for those severe cases, the chances of death remain elevated [2,3]. Factors such as social and psychological stress, economic hardship, and inconsistent virulence of SARS-CoV-2 are likely contributing to the apparent lack of adherence to the advised behavior modifications. The ability to identify those individuals whose health is most at risk by SARS-CoV-2 may allow society to balance social re-engagement more efficiently with protection of public health. School attendance, mass gatherings, travel, and so forth, may be able to resume more fully.

Despite some suggestions, there are no drugs available to cure the patients affected by this catastrophic disease [4]. The identification of effective medicines to fight this disease is urgently needed. Existing host-directed therapies, which have proven to be safe, were suggested to help fight COVID-19 infections [5,6,7]. To identify more therapeutic drugs, we focused on a special G-protein-coupled receptor (GPCR) family named type 2 taste receptors (T2Rs), which was shown to play a critical role in host defense pathways [8,9]. Originally, T2Rs, whose ligands are bitter substances, were thought to be only expressed in the tongue. However, recent studies revealed that they are widely expressed in extraoral tissues, such as the central nervous system, respiratory tract, breast, heart, gastric and intestinal mucosa, bladder, pancreas, testes, and others [10,11]. This suggests that T2Rs might have functions other than bitter taste perception. Indeed, they were suggested to be involved in appetite regulation, the treatment of asthma, the regulation of gastrointestinal motility, and the control of innate immunity [9,11,12,13]. T2Rs can be classified into broadly, narrowly, and intermediately tuned receptors according to the agonist spectra [14].

In the airway, T2Rs are present on ciliated epithelial cells and solitary chemosensory cells (SCC). T2R38, one of the many isoforms of T2Rs, is a receptor that is localized to motile cilia in humans, and is agonized by phenylthiocarbamide (PTC) and propylthiouracil (PROP) [15]. When T2R38 is stimulated by agonists, nitric oxide (NO) is produced to increase mucociliary clearance (MCC) and kill pathogens in the human respiratory mucosa [16].

Interestingly, in a prior study evaluating the effects of NO on SARS-CoV, Åkerström et al. found that NO inhibits the replication of SARS-CoV by two distinct mechanisms. Firstly, NO or its derivatives cause a reduction in the palmitoylation of nascently expressed spike (S) protein which affects the fusion between the S protein and its cognate receptor, angiotensin-converting enzyme 2 (ACE2). Secondly, NO or its derivatives cause a reduction in viral RNA production in the early steps of viral replication, and this could possibly be due to an effect on one or both of the cysteine proteases encoded in Orf1a of SARS-CoV [17].

Three single nucleotide polymorphisms in the gene that encodes T2R38, TAS2R38, confer two common haplotypes including the functional variant PAV (proline–alanine–valine) and the nonfunctional variant AVI (alanine–valine–isoleucine). Homozygotes for the functional allele (PAV/PAV) perceive T2R38 agonists like PTC and PROP as intensely bitter, while homozygotes for the nonfunctional allele (AVI/AVI) are unable to perceive this bitterness. Heterozygotes (PAV/AVI) demonstrate a wide range of bitter taste perception depending on the level of expression of the nonfunctional and functional alleles [18,19]. The homozygotes for the functional alleles, nonfunctional alleles, and heterozygotes were classified as supertasters, nontasters, and tasters, respectively. Sinonasal epithelial cells cultured from AVI/AVI individuals compared to cells cultured from PAV/PAV individuals also demonstrate reduced NO release with a resultant decrease in ciliary beat frequency (CBF) and MCC. Compared to PAV/PAV CRS patients, AVI/AVI patients also demonstrate increased susceptibility to upper respiratory infections [20,21]. 

Prior studies have shown evidence for an association between the PTC/PROP taste test and sinonasal innate immunity, concluding that the ability to assess airway taste receptor variation with an inexpensive taste test has broad implications, as differences in airway taste receptor function may reflect impaired innate immunity and predisposition to certain respiratory infections and inflammatory disorders, and T2R38 functionality in the tongue correlates with nasal symptoms in healthy individuals [22,23].

In a retrospective study performed by Barham et al. on 100 positive cases of COVID-19 confirmed by polymerase chain reaction (PCR), phenotypic expression of T2R38 with taste strip testing appeared to associate with the clinical course and symptomatology specific to each individual as 100% of the patients requiring inpatient admission were classified as nontasters. Conversely, supertasters represented 0% of the patient population, suggesting the possibility of innate immunity to SARS-CoV-2 [1].

As previously mentioned, T2Rs in the upper airway are not limited to ciliated epithelial cells, but are also on solitary chemosensory cells (SCCs), which are rare, nonciliated, epithelial cells which express both sweet (T1R2/3) and T2R receptors. While acyl-homoserine lactones (AHLs) in the human nose stimulate T2Rs on ciliated cells to activate NO production, in vitro studies have found that activation of T2Rs present on human SCCs by denatonium benzoate (DB) and other bitter-tasting compounds such as absinthin, parthenolide, and amoraogentin results in a release of intracellular Ca^2+^, which propagates to the surrounding epithelial cells via gap junctions and stimulates release of antimicrobial peptides(AMPs) stores [16]. AMPs include β-defensin-1 and 2 in the epithelial cells of the respiratory tract that can vigorously block the interaction between the virus and its receptor. Significantly, this immune activation does not occur with AHL stimulation of human SCCs. It is hypothesized that an as yet unidentified bacterial product/byproduct triggers T2Rs on human SCCs to activate this robust antimicrobial defense pathway [24]. Markogenin et al. found that the stimulation of T2Rs on SCC via DB resulted in inhibition of human respiratory epithelial two-pore potassium current in polarized nasal epithelial cells (via a cAMP-dependent signaling pathway), leading to lower threshold for human β-defensin-2 release [25].

One proposed hypothesis suggested that any bitter-tasting drug could have some unintended effects in the body through the activation of T2Rs [26]. With the widespread distribution of the approximately 25 T2Rs in human tissues, inhaled or orally administered bitter drugs could also exhibit off-target effects that are beneficial to the system [27]. There are few reports of bitter-tasting antibiotics activating T2Rs. Ofloxacin has been shown to activate T2R9, and chloramphenicol and erythromycin activate multiple T2Rs [19,28]. Analysis of the structural features of antibiotics from classes including fluoroquinolones, aminoglycosides, and macrolides reveals their close identity with or as derivatives of the parent structures of the aforementioned bitter-tasting compounds. Thus, many of the prescribed antibiotics might also interact with T2Rs expressed in the extraoral tissues [29].

In a prior study, Jaggupilli et al. performed experiments to determine the bitterness of the antibiotics by electronic taste sensor analysis or electronic tongue (E-tongue) analysis. The E-tongue does not contain actual taste receptors; it predicts the taste of test compounds in reference to known compounds based on physiochemical properties and conductivity measurements. It is commonly used to predict the taste of pharmaceutical formulations including antibiotics which might be harmful. The data for the antibiotics tested presented different ranges of predicted bitterness score, with a high predicted bitterness for the azithromycin (15.8), and lower bitterness scores for levofloxacin (4.5) and tobramycin (3.5). Interestingly, the antibiotic with the highest bitterness score, azithromycin, activated T2R4 [29].

Quinine derivatives bind to the T2Rs (expressed in SCCs) and airway smooth muscle cells [30] with the resultant stimulation of airway smooth muscle cells leading to airway relaxation [31]. Chloroquine (CQ) has been tested in a prophylactic and treatment model of allergic airways disease (murine asthma) and was able to mitigate airway inflammation, remodeling, mucus secretion, and airway hyperresponsiveness, some of the cardinal features of allergen-induced asthma in mice [32]. CQ has been shown to have an antimitogenic effect on airway smooth muscle, inhibiting the growth of human airway smooth muscle cells by activating T2Rs [33], and it may provide additional beneficial effects particularly as an immunomodulator [32,34].

Based on the aforementioned, we proposed a treatment protocol for COVID-19 patients based on their T2R38 phenotype (supertasters, tasters, and nontasters) dependent on the fact that supertasters have two copies of the functional alleles (PAV/PAV) and should not require agonists to their T2Rs, as they have high levels of NO to eliminate infection. On the other hand, tasters (those with one functional allele; PAV/AVI) would require a T2R agonist to boost their NO levels. That is why we proposed the supplying of azithromycin, not only as an anti-inflammatory drug, but also as a T2R agonist. The same protocol is provided for the nontaster (T2R38) group, but for a longer duration. Hydroxychloroquine (HCQ) was used in tasters as quinine derivatives, which are known agonists of T2Rs. Dexamethasone was added to all three groups to limit their nasal inflammations, congestion, and cytokine storm, and assist in olfaction preservation. 

## 2. Methods

From our dataset of subjects who were phenotypically tested for T2R38, we included 747 COVID-19 patients who tested positive for SARS-CoV-2 (via PCR) to create this prospective study by providing treatment protocols to COVID-19 patients based on their T2R38 phenotypic expression (supertasters, tasters, and nontasters). This study was conducted in our tertiary referral center from July 2020 till the end of December 2020. The taste test was performed prior to infection with COVID-19.

Phenotype expression of T2R38 was evaluated via taste strip tests to evaluate the genetically determined taste response phenotype of each subject. This study used an early prototype general wellness test kit, being developed along with a software function and now owned by Phenomune LLC (The Woodlands, TX, USA) designed to be used by persons at home to detect, interpret, record, and produce a trait report describing a person’s unique intensity level of phenotypic expression of bitter taste receptors, intended to increase a person’s awareness of his/her sensitivity to bitter tastes for general improvement to functions associated with a general state of health, such as healthy lifestyle choices to enable wellness monitoring as it relates to dietary choices. There are several other commercially available taste strip tests, such as ones sold by Bartovation and Eisco Labs; however, any earlier prototype test kit consistent with the Phenomune general wellness test kit was used by the investigator in this study due to its proprietary interpretation system for determining the scaled intensity of expression in order to facilitate a more precise classification of each subject. These taste strip tests included Control (chemical-free), PTC, Thiourea, and Sodium Benzoate.

Demonstration and Interpretation of the Taste Strip Test

All subjects were presented with the taste test strips with the following order:

1. Control strip
2. PTC strip
3. Thiourea strip
4. Sodium Benzoate strip

Subjects were instructed to place the provided litmus paper strip on their tongue until completely moistened, then the next litmus paper strip was provided according to the order stated above. Subjects were instructed to comment on the quality of taste they perceived, and in addition, to comment on its intensity on a visual analog scale from 0 to 10, where 0 was no perception and 10 was extremely intense quality perceived as compared to the control paper. Each participant was oriented to the scale with a verbal explanation prior to proceeding. In between each taste strip provided, subjects were allowed to sip water. 

**Figure 1:**
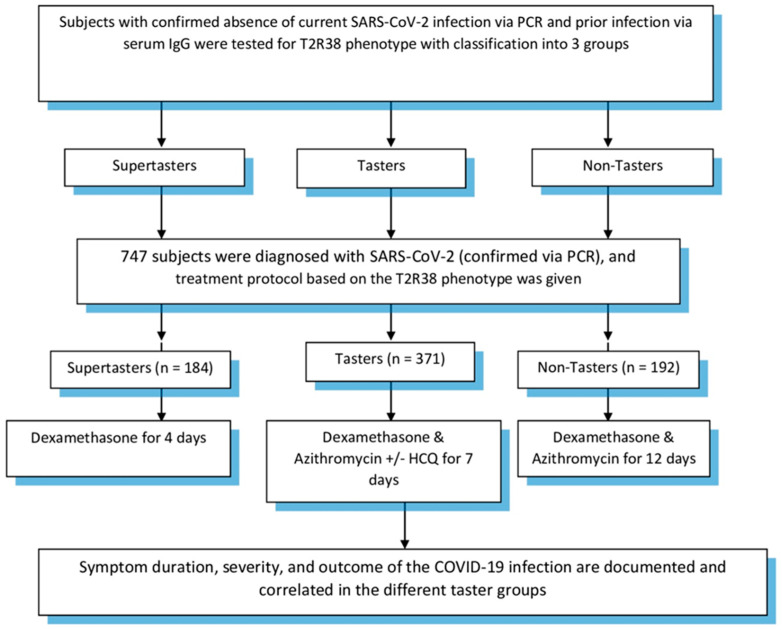
Study design.

Our proposed treatment protocol for COVID-19 patients included dexamethasone for supertasters for four days. Tasters, on the other hand, were given azithromycin, dexamethasone +/− hydroxychloroquine (based on symptom severity of fevers and shortness of breath with oxygen desaturation) for seven days. Finally, nontasters were given azithromycin and dexamethasone for a 12-day duration. Patients were followed up until the end point of our study, which was patient recovery in the form of a negative PCR and/or presence of IgG in their serum samples and/or absence of symptoms. In all three groups, the respective treatment protocol was started within 72 h of symptom onset with a positive PCR for SARS-CoV-2. Patients were reassessed at completion of their treatment protocol and symptomatic treatment was offered when necessary. Study design is demonstrated in Figure 1. Subject inclusion was approved by the Baton Rouge General Institutional Review Board (IRB00005439).

## 3. Exclusions

Subjects with evidence of active infection with SARS-CoV-2 via PCR at study commencement were excluded. Subjects with evidence of prior infection with SARS-CoV-2 via IgG at study commencement were excluded. Subjects with positive results to the Control strip were excluded from evaluation.

Statistical analyses were performed using SPSS v 22 (SPSS Statistics for Windows, version 22.0; IBM, Armonk, NY, USA). Descriptive data are presented as percentages and means ± standard deviation (SD). The correlation coefficient (ICC) was used to determine inverse linear correlation between age and phenotype. Kendall’s tau-B was used for ordinal values. Chi-squared analysis was used for relationships of nominal variables. The prevalence of age and related comorbidities was also compared across treatment cohorts using Pearson chi-square analysis for relationships of nominal variables. Student t test (two-tailed) was used for comparisons of parametric data. Results were deemed significant with a *p* value of <0.05.

## 4. Results

**Figure 2:**
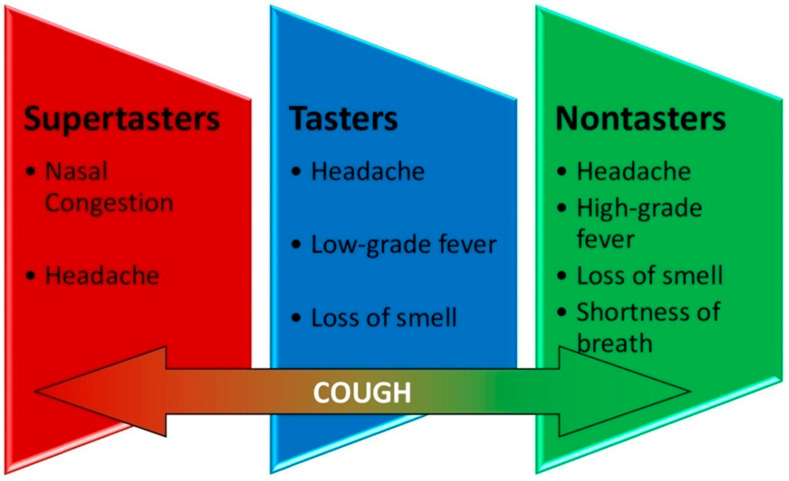
Most common symptoms in each group. Cough was a common variant for all groups.

Seven hundred forty-seven (423 (56.6%) femalewith a mean (SD) age of 46.3 (13.7) years) COVID-19 patients (diagnosed by PCR) were included in our study, commencing July 2020 through the end of December 2020 (Table 1). One hundred eighty-four supertasters (24.7%), 371 tasters (49.6%), and 192 nontasters (25.7%) were categorized. Targeted treatment protocol showed significant correlation (*p* < 0.05) based on patients’ T2R38 phenotypic expression. Average age for the supertasters, tasters, and nontasters were 41.1, 46.5, and 50.2, respectively. Average duration of symptoms with this treatment protocol was 5 days for supertasters, 8.1 days for tasters, and 16.2 days for nontasters. Three (0.4%) patients (all nontasters) required hospitalization. Among the 747 positive COVID-19 cases, the main symptoms were fever, nasal congestion, cough, shortness of breath, loss of smell, and headache. Disaggregation of symptoms in the different groups showed that the supertasters’ most common complaints were nasal congestion and headache, while tasters complained mostly of low-grade fever (<100.5 °F), headache, and loss of smell, and finally, nontasters’ main symptoms were the same as tasters but more severe and for a longer duration along with shortness of breath. All groups complained of cough with varying degree (Figure 2). The most common comorbidities were diabetes, hypertension, rhinosinusitis, asthma, and cardiac disease.

## 5. Discussion

In the extraoral airway, bitter receptors do not modulate taste sensation. In this setting, these receptors are present on a variety of cell types with T2Rs present on epithelial ciliated cells, which play a role in innate immune defense when they bind to their specific agonists. Bitter taste perception is mediated by a family of approximately 25 T2Rs, which respond to a variety of bitter compounds such as PTC, denatonium benzoate, strychnine, quinine, and caffeine [35,36].

The innate immune responses elicited via activation of T2R38 include Ca^2+^-driven NO production. NO induces damage to the intracellular components of infectious microbes and, via its action on protein kinase G and guanylyl cyclase, increases CBF, thereby increasing MCC [37,38,39,40]. This increase in CBF accelerates the removal of mucus-trapped pathogens and the dispersion of other antimicrobial compounds produced in response to pathogens [41,42]. Beyond T2R38, recent studies have investigated other T2Rs including T2R4, -14, -16, -41 with similar findings of ubiquitous expression in human ciliated sinonasal epithelium and a bitter ligand-dependent, Ca^2+^-mediated NO production [43,44]. 

These immunoprotective mechanisms are triggered by the recognition of microbial pathogens, which occurs via activation of several receptor types. It was found that T2Rs were able to recognize pathogens and elicit downstream responses within a matter of minutes, unlike the toll-like receptors that recognize the conserved pathogen-associated molecular patterns and take up to 12 h to elicit a downstream gradual immune response due to changes in gene expression. The mechanism by which this response occurs in the sinonasal epithelium has been a topic of investigation for the past decade [45].

Many of the antibiotics are known to have secondary roles influencing host immune responses and regulating physiologic pathways, including macrolides that can regulate inflammatory conditions; for this reason, they are gaining importance in the treatment of asthma [46,47]. They also target immune cells such as leukocytes, decrease the production of proinflammatory cytokines, increase the release of anti-inflammatory cytokines by inhibiting the cytochrome P450 system, and improve macrophage functions such as chemotactic and phagocytic capacity in addition to their primary roles in targeting bacteria. So, the T2Rs’ activation maybe one of these off-target effects of the bitter-tasting antibiotics, especially azithromycin, the one with the highest bitterness score according to a study by Jaggupilli et al. [48,49].

Ciliated sinonasal epithelial cells are an essential component of the first line of defense in upper airway immunity. Effective MCC requires the coordinated ciliary-driven movement of airway surface liquid, composed of mucus-trapped pathogenic organisms and debris, in order to maintain a healthy sinonasal tract. When MCC is impaired, stasis of sinonasal secretions and resultant local inflammation occur, and can be an inciting factor in increasing susceptibility to infection [38,50,51,52,53].

In particular, T2R4, among other T2Rs, is expressed in structural cells of the human lower airways (such as ciliated epithelial cells and smooth muscle cells) [54,55], as well as in lung inflammatory cells (such as macrophages) [56]. TAS2R mRNAs are expressed in vascular cells (such as human pulmonary artery smooth muscle cells), where they might be involved in the regulation of vascular tone [30,57]. Bitter taste receptors seem to possess many functions in the lung, where they appear to regulate ciliary beating, smooth muscle growth/tone, and the production of inflammatory mediators. Recently, T2Rs were found in middle ear samples and certain T2Rs polymorphisms were correlated with chronic otitis media [58].

Multiple prior studies have evaluated azithromycin (alone or in addition to HCQ) and did not show significant difference between the treatment and placebo groups, yet no prior study has looked into the matter from the taster status prospective; this may answer the question of why some patients have a better course and outcome of illness than others, suggesting protection by innate immunity. As we have different genetic make-up, accordingly, we will have different responses to COVID-19, similar to what is found with other upper respiratory tract infections, like influenza. 

Interestingly, the possible role of quinine derivatives in combating the virus has been investigated, with a study [59] stating that the possible mechanism of action of quinine derivatives is to change the acidic conditions of organelles in mammalian cell culture studies [60], as well as to inhibit the terminal glycosylation of ACE2 in vitro against SARS-CoV [61], indicating its possible role in preventing the fusion of the virus with the cell membrane and thus blocking SARS-CoV-2 infection. Quinine derivatives are known agonists of T2Rs.

In our study, the effects of azithromycin showed a correlation to the taster status (measured in accordance withT2R38 phenotype), yet azithromycin is not known to be a T2R38 agonist; however, the study by Jaggupilli et al. showed the highest bitterness score recorded via E-tongue, finding that it activated only T2R4. What caused azithromycin to help reduce the deterioration of tasters and nontasters in our study maybe unknown, and may be related to its effect on T2R4, or other T2Rs, or a connection between the different T2Rs, in which the activation of one T2R causes a downstream signaling to activate other T2Rs. This is an important finding in the midst of a pandemic, associated with many unknowns, detected in a large sample size.

We detected an inverse relationship between age and the T2R38 phenotypic expression. This finding is in agreement with prior studies confirming this relationship [62,63,64,65]. We also observed that in supertasters with a high level of T2R38 phenotype expression, symptoms were more likely to be localized in the upper respiratory tract without systemic involvement. On the other hand, tasters, with a moderate level of T2R38 allelic expression, displayed localized symptoms with the loss of smell and initiation of some generalized symptoms such as low-grade fever. Lastly, for nontasters, the symptoms appeared to be more severe and more generalized than their taster counterparts. Nontasters do not express T2R38 alleles, leading to more systemic infection with generalized symptoms when infected with upper respiratory infections. The localized symptoms with shorter duration in supertasters may be explained by the local battle between the innate immunity and SARS-CoV-2 in the upper respiratory tract, and when the level of T2R38 allelic expression declines, the subject will be more likely to develop more systemic and severe symptoms. In other words, the innate immunity, in the form of T2R38, seems to act as a protective gate in the face of the incoming upper respiratory tract pathogens. 

Another question is whether phenotypic expression of T2R38 can justify the effects of bitter-tasting compounds in COVID-19 patients based on their taster status, and whether T2R38 may be used as a surrogate to other T2Rs, and act as a representative of the innate immunity of the respiratory tract. In other words, does phenotypic expression of T2R38 serve as a surrogate to other T2Rs’ phenotypic testing? This question seems legitimized, especially with the prior study of Barham et al. [1] showing significant correlation of T2R38 with COVID-19 severity.

Based on the literature, this is the first clinical study evaluating treatment protocols of COVID-19 patients based on the T2R38 phenotype. Some limitations and unknowns existed in our study, one of which is the exact physiological route that these bitter compounds implemented in order to demonstrate the significant response observed in our study subjects. Another one was why some studies have exhibited promising results with these bitter compounds while others displayed none. Maybe if we directed these treatment protocols in the pathway of T2Rs and their different phenotypic expressions in COVID-19 patients, we may be offered a definitive answer to this question. One common factor is the bitter taste of these compounds, which may result inT2Rs agonists as an off-target effect. A recent study on ivermectin (a broad-spectrum antiparasitic drug with extremely bitter taste) against SARS-CoV-2 under in vitro conditions revealed that it can inhibit the viral replication. The single treatment of this drug was able to reduce the virus up to 5000-fold in culture within 48 h [66]. The mechanism by which ivermectin responded against the COVID-19 virus is unknown. Another matter is that the different responses of subjects to COVID-19 are likely related to their genetic make-up. Based on the bitter taste of these compounds, we proposed that the different responses of the subjects in different studies of COVID-19 are dependent on their genotype of the T2Rs receptors.

## 6. Conclusions

With the wide distribution of T2Rs in the respiratory tract, and multiple treatment protocols offered worldwide showing nonconclusive results regarding their effectiveness, this study suggests that treatment protocols for COVID-19 may be modified based on T2Rs phenotypic expression to achieve success in treating COVID-19, and possibly many other respiratory tract pathogens.

## Figures and Tables

**Table 1:** Demographics.

COVID-19 Patients (n = 747)				
Variable	All	Supertasters	Tasters	Nontasters
Number	747	184	371	192
Age	46.3 (13.7)	41.1 (11.0)	46.5 (13.7)	50.2 (15.8)
Sex (F)	423 (56.6%)	122 (66.3%)	189 (50.9%)	112 (58.3%)
Duration of symptoms		5	8.1	16.2
Most common symptoms		Nasal congestion Headache	Low-grade fever Headache Loss of smell	High-grade fever Headache Loss of smell Shortness of breath

## Data Availability

Not applicable.

## References

[1] Barham H.P., Taha M.A., Hall C.A. (2020). Does phenotypic expression of bitter taste receptor T2R38 show association with COVID-19 severity?. Int. Forum Allergy Rhinol..

[2] Bassetti M., Vena A., Giacobbe D.R. (2020). The novel Chinese coronavirus (2019-nCoV) infections: Challenges for fighting the storm. Eur. J. Clin. Investig..

[3] Parry J. (2020). Wuhan: Britons to be evacuated as scientists estimate 44 000 cases of 2019-nCOV in the city. BMJ.

[4] Lu H. (2020). Drug treatment options for the 2019-new coronavirus (2019-nCoV). Biosci. Trends.

[5] Zumla A., Hui D.S., Azhar E.I., Memish Z.A., Maeurer M. (2020). Reducing mortality from 2019-nCoV: Host-directed therapies should be an option. Lancet.

[6] Zumla A., Chan J.F.W., Azhar E.I., Hui D.S.C., Yuen K.-Y. (2016). Coronaviruses—Drug discovery and therapeutic options. Nat. Rev. Drug Discov..

[7] Rao M., Dodoo E., Zumla A., Maeurer M. (2019). Immunometabolism and Pulmonary Infections: Implications for Protective Immune Responses and Host-Directed Therapies. Front. Microbiol..

[8] Li D., Zhang J. (2014). Diet Shapes the Evolution of the Vertebrate Bitter Taste Receptor Gene Repertoire. Mol. Biol. Evol..

[9] Avau B., Depoortere I. (2015). The bitter truth about bitter taste receptors: Beyond sensing bitter in the oral cavity. Acta Physiol..

[10] Lu P., Zhang C.-H., Lifshitz L.M., Zhuge R. (2017). Extraoral bitter taste receptors in health and disease. J. Gen. Physiol..

[11] Lee R.J., Cohen N.A. (2015). Taste receptors in innate immunity. Cell. Mol. Life Sci..

[12] Cohen N.A. (2017). The genetics of the bitter taste receptor T2R38 in upper airway innate immunity and implications for chronic rhinosinusitis. Laryngoscope.

[13] Luo X.-C., Chen Z.-H., Xue J.-B., Zhao D.-X., Lu C., Li Y.-H., Li S.-M., Du Y.-W., Liu Q., Wang P. (2019). Infection by the parasitic helminth Trichinella spiralis activates a Tas2r-mediated signaling pathway in intestinal tuft cells. Proc. Natl. Acad. Sci. USA.

[14] Behrens M., Meyerhof W. (2013). Bitter taste receptor research comes of age: From characterization to modulation of TAS2Rs. Semin. Cell Dev. Biol..

[15] Kim U.K., Drayna D. (2004). Genetics of individual differences in bitter taste perception: Lessons from the PTC gene. Clin. Genet..

[16] Lee R.J., Kofonow J.M., Rosen P.L., Siebert A.P., Chen B., Doghramji L., Xiong G., Adappa N.D., Palmer J.N., Kennedy D.W. (2014). Bitter and sweet taste receptors regulate human upper respiratory innate immunity. J. Clin. Investig..

[17] Åkerström S., Gunalan V., Keng C.T., Tan Y.-J., Mirazimi A. (2009). Dual effect of nitric oxide on SARS-CoV replication: Viral RNA production and palmitoylation of the S protein are affected. Virology.

[18] Bufe B., Breslin P.A.S., Kuhn C., Reed D.R., Tharp C.D., Slack J.P., Kim U.-K., Drayna D., Meyerhof W. (2005). The molecular basis of individual differences in phenylthiocarbamide and propylthiouracil bitterness perception. Curr. Biol..

[19] Meyerhof W., Batram C., Kuhn C., Brockhoff A., Chudoba E., Bufe B., Appendino G., Behrens M. (2010). The Molecular Receptive Ranges of Human TAS2R Bitter Taste Receptors. Chem. Senses.

[20] Adappa N.D., Truesdale C.M., Ba A.D.W., Rn L.D., Mansfield C., Kennedy D.W., Palmer J.N., Cowart B.J., Cohen N.A. (2016). Correlation of T2R38 taste phenotype and in vitro biofilm formation from nonpolypoid chronic rhinosinusitis patients. Int. Forum Allergy Rhinol..

[21] Rom D., Christensen J., Alvarado R., Sacks R., Harvey R. (2017). The impact of bitter taste receptor genetics on culturable bacteria in chronic rhinosinusitis. Rhinol. J..

[22] Farquhar D.R., Kovatch K.J., Palmer J.N., Shofer F.S., Adappa N.D., Cohen N.A. (2014). Phenylthiocarbamide taste sensitivity is associated with sinonasal symptoms in healthy adults. Int. Forum Allergy Rhinol..

[23] Workman A.D., Brooks S.G., Kohanski M.A., Blasetti M.T., Cowart B.J., Mansfield C., Kennedy D.W., Palmer J.N., Adappa N.D., Reed D.R. (2018). Bitter and sweet taste tests are reflective of disease status in chronic rhinosinusitis. J. Allergy Clin. Immunol. Pract..

[24] Douglas J.E., Cohen N.A. (2017). Taste Receptors Mediate Sinonasal Immunity and Respiratory Disease. Int. J. Mol. Sci..

[25] Kohanski M.A., Brown L., Orr M., Tan L.H., Adappa N.D., Palmer J.N., Rubenstein R.C., Cohen N.A. (2021). Bitter taste receptor agonists regulate epithelial two-pore potassium channels via cAMP signaling. Respir. Res..

[26] Clark A.A., Liggett S.B., Munger S.D. (2012). Extraoral bitter taste receptors as mediators of off-target drug effects. FASEB J..

[27] Levit A., Nowak S., Peters M., Wiener A., Meyerhof W., Behrens M., Niv M.Y. (2013). The bitter pill: Clinical drugs that activate the human bitter taste receptor TAS2R14. FASEB J..

[28] Dotson C.D., Zhang L., Xu H., Shin Y.-K., Vigues S., Ott S.H., Elson A.E.T., Choi H.J., Shaw H., Egan J.M. (2008). Bitter Taste Receptors Influence Glucose Homeostasis. PLoS ONE.

[29] Jaggupilli A., Singh N., De Jesus V.C., Gounni M.S., Dhanaraj P., Chelikani P. (2018). Chemosensory bitter taste receptors (T2Rs) are activated by multiple antibiotics. FASEB J..

[30] Manson M.L., Säfholm J., Al-Ameri M., Bergman P., Orre A.-C., Swärd K., James A., Dahlén S.-E., Adner M. (2014). Bitter taste receptor agonists mediate relaxation of human and rodent vascular smooth muscle. Eur. J. Pharmacol..

[31] Pulkkinen V., Manson M.L., Säfholm J., Adner M., Dahlén S.-E. (2012). The bitter taste receptor (TAS2R) agonists denatonium and chloroquine display distinct patterns of relaxation of the guinea pig trachea. Am. J. Physiol. Cell. Mol. Physiol..

[32] Sharma P., Yi R., Nayak A.P., Wang N., Tang F., Knight M.J., Pan S., Oliver B., Deshpande D.A. (2017). Bitter Taste Receptor Agonists Mitigate Features of Allergic Asthma in Mice. Sci. Rep..

[33] Sharma P., Panebra A., Pera T., Tiegs B.C., Hershfeld A., Kenyon L.C., Deshpande D.A. (2016). Antimitogenic effect of bitter taste receptor agonists on airway smooth muscle cells. Am. J. Physiol. Cell. Mol. Physiol..

[34] McAlinden K.D., Deshpande D.A., Ghavami S., Xenaki D., Sohal S.S., Oliver B.G., Haghi M., Sharma P. (2019). Autophagy Activation in Asthma Airways Remodeling. Am. J. Respir. Cell Mol. Biol..

[35] Brockhoff A., Behrens M., Massarotti A., Appendino G.B., Meyerhof W. (2007). Broad Tuning of the Human Bitter Taste Receptor hTAS2R46 to Various Sesquiterpene Lactones, Clerodane and Labdane Diterpenoids, Strychnine, and Denatonium. J. Agric. Food Chem..

[36] Hansen J.L., Reed D.R., Wright M.J., Martin N.G., Breslin P.A.S. (2006). Heritability and Genetic Covariation of Sensitivity to PROP, SOA, Quinine HCl, and Caffeine. Chem. Senses.

[37] Lee R.J., Xiong G., Kofonow J.M., Chen B., Lysenko A., Jiang P., Abraham V., Doghramji L., Adappa N.D., Palmer J.N. (2012). T2R38 taste receptor polymorphisms underlie susceptibility to upper respiratory infection. J. Clin. Investig..

[38] Parker D., Prince A. (2011). Innate Immunity in the Respiratory Epithelium. Am. J. Respir. Cell Mol. Biol..

[39] Zhang Y., Hoon M.A., Chandrashekar J., Mueller K.L., Cook B., Wu D., Zuker C.S., Ryba N.J.P. (2003). Coding of sweet, bitter, and umami tastes: Different receptor cells sharing similar signaling pathways. Cell.

[40] Iwata S., Yoshida R., Ninomiya Y. (2014). Taste transductions in taste receptor cells: Basic tastes and moreover. Curr. Pharm. Des..

[41] Sollai G., Melis M., Pani D., Cosseddu P., Usai I., Crnjar R., Bonfiglio A., Barbarossa I.T. (2017). First objective evaluation of taste sensitivity to 6-n-propylthiouracil (PROP), a paradigm gustatory stimulus in humans. Sci. Rep..

[42] Salathe M. (2007). Regulation of Mammalian Ciliary Beating. Annu. Rev. Physiol..

[43] Hariri B.M., McMahon D.B., Chen B., Freund J.R., Mansfield C.J., Doghramji L.J., Adappa N.D., Palmer J.N., Kennedy D.W., Reed D.R. (2017). Flavones modulate respiratory epithelial innate immunity: Anti-inflammatory effects and activation of the T2R14 receptor. J. Biol. Chem..

[44] Yan C.H., Hahn S., McMahon D., Bonislawski D., Kennedy D.W., Adappa N.D., Palmer J.N., Jiang P., Lee R.J., Cohen N.A. (2017). Nitric Oxide Production is Stimulated by Bitter Taste Receptors Ubiquitously Expressed in the Sinonasal Cavity. Am. J. Rhinol. Allergy.

[45] Hume D.A., Underhill D.M., Sweet M.J., Ozinsky A.O., Liew F.Y., Aderem A. (2001). Macrophages exposed continuously to lipopolysaccharide and other agonists that act via toll-like receptors exhibit a sustained and additive activation state. BMC Immunol..

[46] Culic O., Erakovic V., Parnham M.J. (2001). Anti-inflammatory effects of macrolide antibiotics. Eur. J. Pharmacol..

[47] Good J.T., Rollins D.R., Martin R.J. (2012). Macrolides in the treatment of asthma. Curr. Opin. Pulm. Med..

[48] Rollins D.R., Beuther D.A., Martin R.J. (2010). Update on Infection and Antibiotics in Asthma. Curr. Allergy Asthma Rep..

[49] Gao X., Ray R., Xiao Y., Ishida K., Ray P. (2010). Macrolide antibiotics improve chemotactic and phagocytic capacity as well as reduce inflammation in sulfur mustard-exposed monocytes. Pulm. Pharmacol. Ther..

[50] Sleigh M.A., Blake J.R., Liron N. (1988). The propulsion of mucus by cilia. Am. Rev. Respir. Dis..

[51] Kato A., Schleimer R.P. (2007). Beyond inflammation: Airway epithelial cells are at the interface of innate and adaptive immunity. Curr. Opin. Immunol..

[52] Patel N.N., Kohanski M.A., Maina I.W., Bs V.T., Bs A.D.W., Tong C.C., Kuan E.C., Bosso J.V., Adappa N.D., Palmer J.N. (2018). Solitary chemosensory cells producing interleukin-25 and group-2 innate lymphoid cells are enriched in chronic rhinosinusitis with nasal polyps. Int. Forum Allergy Rhinol..

[53] Kohanski M.A., Workman A.D., Patel N.N., Hung L.-Y., Shtraks J.P., Chen B., Blasetti M., Doghramji L., Kennedy D.W., Adappa N.D. (2018). Solitary chemosensory cells are a primary epithelial source of IL-25 in patients with chronic rhinosinusitis with nasal polyps. J. Allergy Clin. Immunol..

[54] Deshpande D.A., Wang W.C.H., McIlmoyle E.L., Robinett K.S., Schillinger R.M., An S.S., Sham J.S.K., Liggett S.B. (2010). Bitter taste receptors on airway smooth muscle bronchodilate by localized calcium signaling and reverse obstruction. Nat. Med..

[55] Shah A.S., Ben-Shahar Y., Moninger T.O., Kline J.N., Welsh M.J. (2009). Motile Cilia of Human Airway Epithelia Are Chemosensory. Science.

[56] Grassin-Delyle S., Abrial C., Brollo M., Fayad-Kobeissi S., Naline E., Devillier P. (2014). Characterization of the expression and the role of bitter taste receptors in human lung parenchyma and macrophages. Am. J. Respir. Crit. Care Med..

[57] Upadhyaya J.D., Singh N., Sikarwar A.S., Chakraborty R., Pydi S.P., Bhullar R.P., Dakshinamurti S., Chelikani P. (2014). Dextromethorphan mediated bitter taste receptor activation in the pulmonary circuit causes vasoconstriction. PLoS ONE.

[58] Kaufman A.C., Colquitt L., Ruckenstein M.J., Bigelow D.C., Eliades S.J., Xiong G., Lin C., Reed D.R., Cohen N.A. (2021). Bitter Taste Receptors and Chronic Otitis Media. Otolaryngol. Neck Surg..

[59] Florindo H.F., Kleiner R., Vaskovich-Koubi D., Acúrcio R.C., Carreira B., Yeini E., Tiram G., Liubomirski Y., Satchi-Fainaro R. (2020). Immune-mediated approaches against COVID-19. Nat. Nanotechnol..

[60] Savarino A., Boelaert J.R., Cassone A., Majori G., Cauda R. (2003). Effects of chloroquine on viral infections: An old drug against today’s diseases. Lancet Infect. Dis..

[61] Vincent M.J., Bergeron E., Benjannet S., Erickson B.R., Rollin P.E., Ksiazek T.G., Seidah N.G., Nichol S.T. (2005). Chloroquine is a potent inhibitor of SARS coronavirus infection and spread. Virol. J..

[62] Mennella J.A., Pepino M.Y., Duke F.F., Reed D.R. (2010). Age modifies the genotype-phenotype relationship for the bitter receptor TAS2R38. BMC Genet..

[63] Mennella J.A., Reed D.R., Roberts K.M., Mathew P.S., Mansfield C.J. (2014). Age-Related Differences in Bitter Taste and Efficacy of Bitter Blockers. PLoS ONE.

[64] Whissell-Buechy D. (1990). Effects of age and sex on taste sensitivity to phenylthiocarbamide (PTC) in the Berkeley Guidance sample. Chem. Senses.

[65] Whissell-Buechy D., Wills C. (1989). Male and female correlations for taster (P.T.C.) phenotypes and rate of adolescent development. Ann. Hum. Biol..

[66] Caly L., Druce J.D., Catton M.G., Jans D.A., Wagstaff K.M. (2020). The FDAapproved Drug Ivermectin inhibits the replication of SARS-CoV-2 in vitro. Antivir. Res..

